# Frontline Health Care Workers’ Mental Health and Well-Being During the First Year of the COVID-19 Pandemic: Analysis of Interviews and Social Media Data

**DOI:** 10.2196/43000

**Published:** 2023-08-14

**Authors:** Norha Vera San Juan, Sam Martin, Anna Badley, Laura Maio, Petra C Gronholm, Caroline Buck, Elaine C Flores, Samantha Vanderslott, Aron Syversen, Sophie Mulcahy Symmons, Inayah Uddin, Amelia Karia, Syka Iqbal, Cecilia Vindrola-Padros

**Affiliations:** 1 Rapid Research Evaluation and Appraisal Lab (RREAL) Department of Targeted Intervention University College London London United Kingdom; 2 Centre for Global Mental Health and Centre for Implementation Science, Health Services and Population Research Department Institute of Psychiatry, Psychology and Neuroscience King’s College London London United Kingdom; 3 Ethox Centre, Big Data Institute University of Oxford Oxford United Kingdom; 4 Academy Research and Improvement Solent Trust Southampton United Kingdom; 5 School of Health Sciences University of Southampton Southampton United Kingdom; 6 Department of Behavioural Science and Health University College London London United Kingdom; 7 Centre on Climate Change & Planetary Health London School of Hygiene and Tropical Medicine London United Kingdom; 8 Stanford Center for Innovation in Global Health, Stanford Woods Institute for the Environment, Stanford University Stanford, CA United States; 9 Oxford Vaccine Group, Churchill Hospital University of Oxford Oxford United Kingdom; 10 NIHR Oxford Biomedical Research Centre University of Oxford Oxford United Kingdom; 11 Institute of Epidemiology and Healthcare University College London London United Kingdom; 12 Centre for Interdisciplinary Research, Education and Innovation in Health Systems, School of Nursing, Midwifery and Health Systems University College Dublin Dublin Ireland; 13 Division of Psychiatry, Marie Curie Palliative Care Research Department University College London London United Kingdom; 14 Department of Psychology University of Bradford Bradford United Kingdom

**Keywords:** mental health, frontline, health care workers, COVID-19, health services research, Collaborative and Digital Analysis of Big Qualitative Data in Time Sensitive Contexts, LISTEN method

## Abstract

**Background:**

The COVID-19 pandemic has shed light on fractures in health care systems worldwide and continues to have a significant impact, particularly in relation to the health care workforce. Frontline staff have been exposed to unprecedented strain, and delivering care during the pandemic has affected their safety, mental health, and well-being.

**Objective:**

This study aimed to explore the experiences of health care workers (HCWs) delivering care in the United Kingdom during the COVID-19 pandemic to understand their well-being needs, experiences, and strategies used to maintain well-being (at individual and organizational levels).

**Methods:**

We analyzed 94 telephone interviews with HCWs and 2000 tweets about HCWs’ mental health during the first year of the COVID-19 pandemic.

**Results:**

The results were grouped under 6 themes: redeployment, clinical work, and sense of duty; well-being support and HCW’s coping strategies; negative mental health effects; organizational support; social network and support; and public and government support.

**Conclusions:**

These findings demonstrate the need for open conversations, where staff’s well-being needs and the strategies they adopted can be shared and encouraged, rather than implementing top-down psychological interventions alone. At the macro level, the findings also highlighted the impact on HCW’s well-being of public and government support as well as the need to ensure protection through personal protective equipment, testing, and vaccines for frontline workers.

## Introduction

### Background

High levels of stress, burnout, and symptoms of poor mental health have been well known among health care workers (HCWs) for several years [1]. Although many health systems include mechanisms to support HCW’s well-being, the COVID-19 pandemic has exacerbated the fractures of systems around the world in relation to protecting their health care workforce, which has garnered increased interest in light of developments from the COVID-19 pandemic. Frontline staff, in particular, have been exposed to unprecedented strains. Delivering care during the pandemic impacted their own and their loved one’s safety, and the complexities of the pressures placed them in often difficult and uncomfortable situations for which they had not necessarily been trained [2-4]. Studies have shown the burden on the physical and mental health of delivering care over long hours under the heat of full personal protective equipment (PPE), the strain of changing guidelines, and the rising rates of infection among HCWs [5-8]. This anxiety was exacerbated by the high rates of hospital deaths and the added responsibility many HCWs felt to accompany patients during the last moments of their life so they would not die alone [9]. Dowrick et al [10] pointed out the significant emotional labor involved in affective practices to mitigate the limitations arising from physical distancing, such as maintaining communication with patient families and keeping in touch with work colleagues who were also going through a difficult time. Although infection control measures were crucial for limiting the spread of COVID-19, they required complex additions to the workload of HCWs to reorganize necessary interactions at work. Our previous work on this topic additionally highlighted how distancing measures completely disrupted environmental factors that influence HCWs’ well-being [4], such as leisure time. Not only does the emotion involved in delivering care under these circumstances add copious amounts of burden to HCWs, but the moral injury of caring for patients under time and resource constraints has also been a frequently mentioned factor associated with poor mental health since the start of the pandemic [11].

In particular, nursing staff have been severely affected by the pandemic [12,13]. Some authors have argued that nursing staff have experienced moral conflicts and complex ethical issues because of the need to make difficult decisions in the context of medical rationing produced by high patient demand and limited resources [14,15]. Nurses have expressed the fear of becoming infected and transmitting the virus to family members [16]. Several studies have reported higher rates of work-related stress among nurses and a higher rate of burnout among nurses when compared with medical staff, while being a woman and a nurse have both been identified as risk factors for poorer mental health outcomes [17,18]. Other risk factors for psychological distress reported in the literature include being younger, being more junior, being the parent of dependent children, having infected family members, or having a frontline role [19-21].

Evidence from previous emergencies, including previous epidemics, has pointed to the detrimental effects of working in these conditions and has highlighted the importance of considering frontline workers’ mental health and well-being [22,23]. During the COVID-19 pandemic, efforts were made to integrate this evidence and learnings from previous epidemics in the development of well-being guidelines and support interventions, but many of these guidelines and interventions focused mainly on the assessment of clinical outcomes, such as the identification of symptoms of posttraumatic stress disorder (PTSD) [24,25]. Emerging evidence shows that social and organizational measures, such as maintaining clear communication, providing adequate PPE to staff, allowing staff to have adequate breaks and rest, and delivering psychological support in a timely and practical way, could help reduce the risk of adverse mental health outcomes and improve the well-being of staff [20,21,24,26].

Well-being refers to an overall state of being encompassing aspects, such as social, physical, and emotional well-being, whereas mental health focuses specifically on psychological health, such as thoughts, beliefs, and behaviors. Well-being and mental health are both interconnected and mutually influence each other [27]. In an early analysis of the mental health needs of HCWs during the COVID-19 pandemic in the United Kingdom, we found that support guidelines did not implement a holistic depiction of well-being. Although some guidelines have recently been expanded to consider the contextual factors that might shape well-being, there is limited evidence on our understanding of the well-being of HCWs (beyond individual mental health) in the context of complex health emergencies, such as the COVID-19 pandemic.

### Objectives

This study conducted an in-depth exploration of the experiences, needs, and well-being strategies of HCWs and institutions delivering care in the United Kingdom during the COVID-19 pandemic. We did not limit our analysis to clinical mental health and defined well-being following the Recovery Model of Social recovery model [25], which considers aspects related to quality of life, protective environments, and support networks. We also explored how these experiences changed during different stages of the pandemic.

## Methods

### Overview

This study is part of a larger ongoing project led by the Rapid Research, Evaluation and Appraisal Lab (RREAL), which was designed as a qualitative rapid appraisal with the aim of analyzing HCWs’ experiences and perceptions of delivering care during the COVID-19 pandemic [7]. Rapid appraisals are developed to collect and analyze data in a targeted and iterative manner within limited timeframes and to “diagnose” a situation. The study combined telephone interviews with a purposive sample of HCWs based in the United Kingdom and an analysis of social media data. These data sources were chosen to combine in-depth information about individual experiences in interviews and the broad range of perspectives present in social media.

### Data Collection

#### Interviews With HCWs

Semistructured interviews were conducted over the phone by members of the RREAL research team, including LM, SM, CVP, AS, and CB. We reported on the findings of 94 interviews conducted between March 2020 and March 2021. The interview topic guide (Multimedia Appendix 1) was revised 4 times during this period, following emerging findings and changing public and policy concerns at the different stages of the pandemic. A detailed description of the data collection and sampling methods can be found in the study by Vera San Juan et al [25].

A group of authors (NVSJ, LM, CB, PCG, AB, AS, ECF, IU, and SMS) prepared interview data for analysis by performing selective transcription of extracts from the interviews and interview notes that were related to mental health and well-being, as previously defined [4]. This team included health service researchers, academics, nurse practitioners, anthropologists, and physicians.

#### Social Media Data

Data were collected from social media accounts of self-identified HCWs in the United Kingdom (Multimedia Appendix 2) between March 19, 2020, and March 19, 2021. Data analysis was guided by the collaborative coding framework analysis provided by the interview analysis. The key themes produced from the interview framework were used to inform metadata and key search terms for social media data collection. This was done after careful initial and replicable analysis of the mental health criteria shared by HCWs from initial interviews. Each theme was broken down into a maximum of 5 bullet-pointed descriptions, and the most prominent keywords were extracted from these thematic summaries added to the advanced Boolean search term (eg, “anxiety” was summarized in the Boolean search term as “anxi*,” so that all variations or spellings of the word “anxiety” would be searched for). HCWs were identified by how they self-identified in their Twitter bios, and we created a sub-Boolean of all possible UK variations of words or descriptions of working in National Health Service (NHS) or hospitals (Multimedia Appendix 2). Therefore, advanced Boolean search terms were created that reflected keywords from the themes in the collaborative coding framework (Multimedia Appendix 3). These Boolean search terms were then used to mine all tweets from Twitter data archives using the media monitoring Meltwater software (2020) for English-language tweets related to the themes found in the interview data (and that were engaged with liked, quoted, and retweeted more than once). An initial data set of 775,000 tweets was sampled from all Twitter posts shared by HCWs in the United Kingdom. Filtering was determined by the advanced Boolean search terms shared in Multimedia Appendix 2. The tweets were then further filtered and cleaned using the inclusion and exclusion criteria during the later social media analysis stage, leaving a final sample of 20,000 English-language tweets.

### Data Synthesis and Analysis

#### Interviews With HCWs

**Figure 1:**
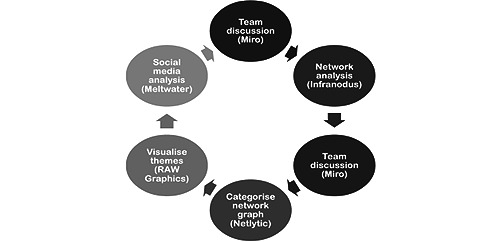
Collaborative and Digital Analysis of Big Qualitative Data in Time Sensitive Contexts analysis cycles. This figure outlines the analysis steps followed and the software used at each step.

The interviews were analyzed by conducting a Collaborative and Digital Analysis of Big Qualitative Data in Time Sensitive Contexts (LISTEN). This analysis consists of iterative cycles of intercalating team discussions and the use of digital text and discourse analytics tools to analyze related social media data (Figure 1).

First, the group of authors who prepared the data for analysis held a guided group discussion (see Collaborative Matrix Analysis [28]) using the Whiteboard Tool Miro (Desktop, version 0.42) to outline (1) the main emerging themes; (2) patterns in the presence of themes in interviews with specific populations or professional groups; and (3) negative cases, which appeared to be out of the norm. On the basis of this, a preliminary coding framework was developed and shared with the digital analysis team (SM and SV) who, using the Infranodus discourse analysis tool [29], developed an analytical coding framework that predicted a preliminary scan of the data, group discussions, and thematic discourse mapping. They conducted a digital analysis of co-occurring themes using betweenness centrality and frequency analysis [30,31] and inputted it into a Microsoft Excel matrix. The framework was refined during team discussions, and further digital inquiries were performed using big qualitative keyword analytics to test sample data with Netlytic [32].

The results from the digital analysis were shared with the research team, and a joint team discussion was held to refine and finalize the coding framework. Codes were defined in a coding book and inputted into a coding matrix in Excel that included codes and participant characteristics in the columns and cases in rows. The researchers completed a matrix for the sets of interviews that they were assigned. NVSJ cross-checked the data during the coding process to ensure consistency across the researchers.

Once data indexing was complete, codes were divided among the team to be synthesized, and selected quotes from the interview transcripts were chosen to exemplify themes. We then held a third team discussion using Miro to assess links between key topics emerging within each code; based on this, we developed the final set of themes encompassing the main issues raised by frontline staff. NVSJ reviewed the definitions of the themes, alongside cross-checking with thematic discourse mapping conducted on Infranodus [29] to ensure consistency in relation to the grouping of codes and the selection of illustrative quotes.

#### Social Media Data

First, tweets were screened to assess data quality and alignment with the inclusion criteria. The process to complete this consisted of four steps: (1) the resulting social media data (tweets) that were collected were then analyzed and coded using the key themes and keywords from the interview-based collaborative framework as a guide; (2) SM and SV selected anonymized tweet examples [33] that could exemplify mental health themes and patterns of discourse in the data; (3) the results from this analysis were then used to structure a comparative social media analysis and sentiment analysis framework (Multimedia Appendix 3); and (4) an Include and Exclude criterion was developed within our social media analysis framework to maintain good data quality, paying attention to the importance of using a mixed methods approach, which involves incorporating both qualitative and quantitative evaluations to assess data quality [34].

After screening for the inclusion and exclusion criteria, 2000 tweets were included in the final analysis. Eight independent coders annotated the tweets via a shared spreadsheet (NV, SM, SV, CB, EF-R, AS, IU, and PG), allocating them to the 5 key themes from the social media analysis coding framework (Multimedia Appendix 3). All coders were of mixed social sciences and public health research abilities, so a simple process of using the method of a shared spreadsheet with separate columns used to annotate tweets for theme and inclusion and exclusion was used. A guide to theme definitions and summarized examples of tweets to include and exclude were also shared in a tab within the spreadsheet. This made it easy for team members less experienced in annotating social media posts to follow, as well as query. After the first initial round of coding, team members met to share their experiences and queries, and it was agreed that tweets were nuanced enough that themes should not be restricted to one theme per post but that more than one theme could be assigned to a post or tweet where that tweet was particularly nuanced. Where there were queries about theme relevance or inclusion and exclusion criteria, each team member marked their annotation with a question mark. Annotation was completed in 2 rounds, where the coders met to discuss and agree on intercoder reliability. After meeting and discussing the tweets that they had queries about, the annotators were able to agree on the coding of all remaining posts. After initial coding, the agreement and reliability of the individual annotators was 71% for the themes (*F*_1_-score 0.62) and 81.4% for the includes and excludes (*F*_1_-score 0.76). These methods of using a shared spreadsheet with separate columns used to annotate tweets for theme and inclusion can easily be repeated by a new team of researchers on another social media data set.

#### Sentiment Analysis

We aimed to obtain a better understanding of the sentiments of tweets within the context of the HCW experience by creating a manual sentiment framework. Drawing on previous research [31,33], we set up our own framework with definitions based on our analysis using a more specific qualitative lens and carefully constructed the qualitative framework. This drew on part 4 of the LISTEN cycle, where we created a sentiment framework that was informed by an analysis of the themes from the qualitative interview coding framework in this study. Sentiment was measured in terms of differences in attitudes toward the impact of mental health on the individual as well as the impact on delivery of care within the context of the pandemic (Multimedia Appendix 4). Posts or articles were classified as positive toward individual mental health experiences of work, such as redeployment; well-being; organizational; social or family; or public or government support on the frontline if, for example, they were affirming of mental health support or experience or communicated overall trust of hospital or government guidelines. Posts were marked as negative if they contained negative attitudes or arguments against the way the mental health of HCWs was supported, shared bad experiences, or discouraged the following of government or hospital guidelines. Posts were then marked as neutral if they contained only a general statement, with no expression of sentiment or opinion. The text of the tweets was analyzed in terms of discourse content, sentiment analysis, and frequency per topic.

#### Discourse Analysis

Thematic discourse mapping was used to analyze the social media data within each of the 5 coding categories (Multimedia Appendix 3) using the software Infranodus [29]. This analysis was used to map co-occurring themes, keyword frequencies, and patterns occurring in the data with regard to discussions about mental health experiences of HCWs, as well as the strength of the betweenness centrality of subthemes (connections between subthemes that link different types of conversation clusters together) [35].

### Ethics Approval

The authors assert that all procedures contributing to this work comply with the ethical standards of the relevant national and institutional committees on human experimentation and with the Helsinki Declaration of 1975, as revised in 2008. This study was approved by the Health Research Authority in the United Kingdom (Integrated Research Application System: 282069) and local research and development offices. All participants provided informed consent before participation.

## Results

### Interview Characteristics

We extracted information relevant to well-being and mental health from 94 interviews with professionals across London, including nurses, physiotherapists, anesthetists, and consultants. We analyzed interviews conducted between March and September 2020, which lasted between 12 minutes and 2 hours (37 min on average). Professionals varied in years of experience and grades, ranging from recently qualified (<1 year) to professionals with over 30 years of experience. Approximately 66% (62/94) of the sample were women, and the average age was 38 (range 22-60) years. A more detailed description of the sample and the interview characteristics can be found in Tables 1-4.

**Table 1:** Demographics of participants (N=94).

Gender	Participants, n (%)
Woman	62 (66)
Man	27 (29)
Not known	5 (5)

**Table 2:** Age demographics of participants.

Age	Values (years)
Minimum	22
Maximum	60
Average	38.2

**Table 3:** Years of experience (N=94).

Years of experience	Professionals, n (%)
0-5	21 (22)
6-10	20 (21)
11-15	13 (14)
16-20	12 (13)
21-25	5 (5)
26-30	7 (7)
31-35	3 (3)
36-40	3 (3)
Unknown	10 (10)

**Table 4:** Ethnicity demographics of participants (N=94).

Ethnicity (macro categories)	Participants, n (%)
Asian or British Asian	7 (7)
Black or Black British	3 (3)
White or White British	36 (38)
Mixed	2 (2)
Not known	46 (48)

### Social Media Data Characteristics

From a total of 775,000 tweets, quoted tweets, and replies, there were 104,000 original tweets, which were quoted or discussed 192,000 times, replied to 364,000 times, and retweeted 443,000 times. A total of 19,000 commented on and quoted tweets or retweets, and 364,000 were replies to the original tweets or retweets. Screening for the inclusion and exclusion criteria (Multimedia Appendix 5) resulted in 2000 tweets, which were included in the final analysis. Comparing the final agreed coding after discussions with the annotators’ initial coding, the accuracy of the individual annotators was 71% for the themes (*F*_1_-score 0.62) and 81.4% for the includes and excludes (*F*_1_-score 0.76).

### Main Themes

#### Overview

The results presented experiences that were perceived by HCWs as affecting their well-being, expressions of well-being needs, and well-being strategies presented by staff. Findings from the analysis of interviews and social media data fell under 6 themes: redeployment, clinical work, and sense of duty; well-being support and HCW’s coping strategies; negative mental health effects; organizational support; social network and support; and public and government support. We described each theme and presented illustrative quotes in the following sections.

#### Redeployment, Clinical Work, and Sense of Duty

Although staff often willingly volunteered to be redeployed, the prospect caused anxiety for a number of HCWs because of speculation that new roles might be entirely outside the normal scope of practice. Some staff reported that options for redeployment were offered via web-based surveys, giving little description of what they were signing up to do. The pace of the crisis required rapid decision-making amid the vague details of what the new role might entail. For team managers, being unable to provide clarity about who would be redeployed and when it was also a cause of distress:

We saw other people were very anxious beforehand and we did a lot of work around trying to support people around those anxieties.

C0V40, consultant pediatrician

New location, unknown faces, difficulty communicating while wearing PPE, and rapidly learning different ways of working were reported as the challenging aspects of navigating redeployment. Concerns about being exposed to the virus were high, and participants informed that there was no risk assessment of susceptible HCWs or those with susceptible family members were undertaken, particularly early on in the pandemic. In addition, typical mechanisms that support a change of job (eg, induction or orientation and management guidance) were limited, increasing the difficulty of day-to-day working. This was particularly true for those redeployed to a role with little relation to their normal job remit (eg, allied health professionals reassigned from outpatient or community settings to working with inpatients in hospital wards):

In terms of just levels of stress, I think for me it’s been very difficult [to] distinguish between not just purely COVID but then also being in a new job, a different role... I know for sure I wouldn’t have felt this stressed had I been in somewhere new [where] I knew the people... I think that’s added a massive amount to me.

COV52, charge nurse

Redeployment was not always voluntary. Some staff reported that they were selected without having much say in the matter, with department managers forced to make the selection. Other participants recounted how an individual’s suitability to a particular discipline was not considered, especially when reassigning a staff member to a complex environment, such as the intensive care unit (ICU):

I think you have some members of staff that were redeployed [who] felt that they were in some way singled out. Why did they have to go? Because it wasn’t necessarily people that volunteered.

COV100, nurse manager

Although a number of participants expressed relief and some pride that they were not redeployed (their roles were deemed important for the maintenance of certain services [eg, surgery, cancer treatment]), some participants expressed feelings of shame to avoid redeployment (intentionally or otherwise) amid the increasing narrative of “heroism” circulated by the media:

Guilty that we were not doing more... that’s why a few of my colleagues volunteered... to do other things in a training capacity or clinical service... that is how we assuaged our guilt.

COV57, anesthetist

Some HCWs who had experienced positive redeployment shared their pride in being able to help but also looked ahead to return to their previous roles:

Today, my redeployment to Covid Intensive Care came to an end. I have never been so proud to have been part of such an incredible workforce that have given and continue to give their absolute all to deliver the best care possible. Next stop > 11th floor in Stroke 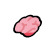.

COVTweet423, physiotherapist

Feelings of resentment were reported as surfacing once some services returned to normal, with the inequity between those redeployed and those who stayed in their normal role becoming apparent, especially if the redeployed staff had not been particularly busy, as was the case for sexual health services:

There were lots of people I think that have now come back that are finding it difficult to fit back into the team. There’s quite a lot of resentment there.

COV100, nurse manager, sexual health

Nurses were perceived by other professionals as enduring more challenges than other disciplines. Although team leads in general expressed that keeping track of team well-being was an important challenge, nurse leads in particular reported struggling to train new staff and manage their physical and emotional well-being while keeping on top of their increased clinical workload:

You have these really frightened inexperienced nurses who sometimes haven’t seen a patient die ever or for a long time who are terrified and immobilized.

COV73, lead nurse

Changes to patient demand and complexity led to many HCWs sharing their discomfort and distress at not being able to provide the level of care and patient experience that they would like and “forced to be comfortable with the uncomfortable” (COV95, profession). Allied health professionals described how they were required to juggle clinical work from their normal role while simultaneously being redeployed to a new position, resulting in neither role being optimally performed:

There was a lot of pressure on us to help everybody... we were finding it difficult ourselves.

COV90, ICU dietician

These feelings of exhaustion were enhanced by the levels of poor patient outcomes and inability to socialize with colleagues. The HCWs described a constant battle between the relentless demand for clinical care and needing to rest:

So much is asked of staff who are already stretched and underpaid. Worn out and stressed staff can’t cope.

COV96, critical care dietician

Overall, the feeling of sadness underpinned many HCWs experiences. This was often related to caring for people who died in the absence of family members. HCWs spoke of absorbing the sadness of dying and the amount of death as well as trying to provide care and consolation to absent relatives remotely:

I think the hardest thing is, is that,...they cannot die alone. That’s so incredibly hard thing to come to terms with, isn’t it? You know, it’s... but, you know, we never allow anyone to die alone, we were always in there with them, because that’s what you do as a nurse.

COV46, infection control nurse

#### Well-Being Support and HCWs Coping Strategies

Most staff believed that the pandemic led to strong leadership from management and facilitated more compassion and awareness to mental health and well-being. Twitter was used as a place to share practical guidelines and well-being resources:

A guide to support managers to strike the right balance between directive and compassionate leadership that will help to ensure their teams come through the #COVID19 crisis with a greater resilience and mutual respect. Both critical to the recovery phase.

COVTweet1269, chief nursing officer

HCWs were offered a range of mental health and well-being supports, including appointments with clinical psychologists, emails with well-being recommendations, and peer support group meetings. HCWs who interacted with psychologists found it useful and expressed their gratitude for the opportunity to be in contact with such professionals. Staff felt it was a privilege to have access to a wide range of support; however, many expressed that it had not been possible to make use of these services. The lack of time due to staff shortages, no holidays, and a sense of duty were often highlighted as barriers for HCWs to access support:

I think the problem is being able to access them on a shift, when it’s just so busy and manic. It’s one thing having the space to go and relax, but if you haven’t got the time to go and do it, then, that’s a different thing.

COV52, ICU nurse

Some HCWs described their experience as “groundhog day-like,” having no sense of progress or direction. Managers and more experienced ICU clinicians were central to providing guidance and support, despite having significant additional clinical work burdens:

Definitely, definitely on sleeping, definitely on mental health because it was constant stress, constant worry, you know as I say as a general manager people would come to me for assurances and I wasn’t able to give the assurances that people were looking for and it was up to me to try and stay calm and support my staff but the same time I felt very little support in return from my superiors and the rest of the organization.

COV117, general manager, critical care

Some managers put in place practices to mitigate this, such as enforcing breaks or being available to talk about things beyond work. This provided some space for staff to briefly focus on their well-being. However, there were dilemmas regarding whether these well-being practices should be mandatory:

I can’t make it mandatory, but I strongly encourage people to have clinical supervision... You’ve got that offer there, you’re an adult, you’re a professional, you have to take some responsibility for your own health and wellbeing.

COV73, lead nurse

Regarding support from peers, infection control rules meant that staff could not socialize in ways they were used both inside and outside of hospitals. HCWs missed informal interactions, particularly staff working remotely, and teams that were broken up due to redeployment. However, most staff working in new environments found it invigorating. Team morale was reported as high, with some people thriving on change, learning new skills, and strengthening relationships and respect amid the difficult times shared. Reduced hierarchy also played into the sense of togetherness and team spirit:

I think there’s been a lot of like solidarity between the teams, trying to get through this and coming together and appreciating the help we’ve had from other disciplines, the redeployed staff, so hopefully they’ve seen how hard we work, and maybe appreciate the efforts of ICU more. I think there’s definitely been a good coming together and an ethos of like we’ve got to try and do our best to get through this.

COV52, ICU charge nurse

Furthermore, some staff felt that they did not need to access support due to their existing knowledge about mental health and the techniques they knew to build resilience. Support from family and friends also played a key role in maintaining a positive mindset. Staff incorporated their own coping strategies in maintaining their well-being, such as using mindfulness apps, performing exercise, and being outdoors, which allowed them to disconnect during times when they felt overwhelmed and exhausted:

Personally feel like I already had enough teaching, and I was prepared for that, it wasn’t hugely beneficial for me, but for other people it was.

COV43, general practitioner trainee

#### Negative Mental Health Effects

The participants described a range of negative mental health effects. Many reported feelings of trauma and PTSD-like symptoms along with a sense of being overwhelmed, emotional, and physical exhaustion. In addition to the distressing factors mentioned earlier, HCWs were affected, for example, by the onslaught of news and negative events occurring to relatives and close friends:

What I was witnessing over the past, in April anyway, was some of the most distressing stuff I’ve ever seen professionally.

COV65, palliative care consultant

HCWs also discussed their experience of caring for patients with COVID-19 in different “COVID zones” within COVID-19 wards (red, orange, green, and blue), whereas some expressed anxiety about ICU capacity, the prolonged amount of time nurses spent working in these wards, and the extreme risks associated with varying access to adequate PPE. Others also reported being in charge of COVID-19 ICUs, where staff were busy, overwhelmed, and depleted by illness, exhaustion, and the need for quarantine. Some HCWs expressed during the height of the pandemic they actively did not stop to consider potential negative effects on their mental well-being. Often, HCWs spoke of potential trauma that was not fully registered until later, at which point many struggled to fully process their experiences and reported feeling less resilient than before:

I think now we’ve got a bit more space to sort of process because I think when it was all happening it was... you just went into almost auto pilot and you couldn’t really emotionally process anything anyway.

COV108, speech and language therapist

“I don’t know whether it’s sort of had an impact on my attention or anything but it’s definitely, erm, I’m feeling more... I don’t feel as robust as I did.”

COV108, speech and language therapist

A key source of anxiety was the threat of becoming infected with COVID-19. This fear was exacerbated by coming into contact with patients with COVID-19, seeing severely unwell and dying patients, and, at the beginning of the pandemic, the lack of understanding of the illness and limited insights regarding possible treatments. Some staff members also spoke of how their anxiety increased over time and the full extent and severity of the pandemic became apparent:

Obviously it makes you fear for yourself and family and friends and those sorts of things and especially with headlines that lots of healthcare workers are you know getting sick and then their dying obviously kinda adds to those concerns as well.

COV69, senior PT

Anxiety was also reported in relation to the fear of inadvertently infecting others. This was a concern, particularly for those who lived with or cared for vulnerable family members, such as older parents. Other sources of anxiety related to COVID-19 infections were concerns for colleagues who became unwell, awareness of the burden placed on family members who worried about frontline staff, and worries about the general public who lacked medical knowledge in dealing with the pandemic. For some, anxiety was brought on by other work-related factors, such as having to give up usual clinical work, lack of control, and not being able to switch off from work. An example of such anxiety was expressed in a tweet by a physician who had worked on the frontline during the first wave of the pandemic:

... extremely vulnerable and sad, and did have a brief mental health relapse. I had to seek help through my GP and saw a psychologist. Why did I become unwell? Stress surrounding lack of PPE, re-deployment, worries about catching COVID-19 and bringing the virus home to my family...

COVTwitter845, physician

Adverse mental health impact was issues with sleep. Racing thoughts, constant exposure to negative events and worries, and an increase in the number of night shifts led to disrupted sleep, which contributed to further exhaustion.

It was a recurring theme where I’d say I’d been awake since four, I couldn’t get back to sleep after I woke up feeling this overwhelming feeling of threat, panic, but not, not like palpitations or breathlessness just an impending doom, that was kind of what it was...

COV95, consultant anesthetist

Another key mental health impact was the feeling of sadness over witnessing a high number of deaths. Participants also spoke of how it was particularly difficult to see the impact of COVID-19 on patients who would normally not be expected to become severely unwell, such as otherwise healthy, younger people:

Some people that stick in my head are patients that were quite young erm, like patients in their thirties or patients in their forties with zero comorbidities coming into it, and then were in the hospital for about fifty days and had trouble weaning from the ventilator.

COV83, PT

Many patients also described concerns about what was ahead. A key source of concern was fear of further COVID-19 waves, after first-hand experience of how challenging the first wave was and how frontline workers were still reeling in its aftermath. Many patients concerns regarding the suspended nonemergency procedures and treatments that still needed to be performed alongside routine care by a jaded workforce. Participants also sensed that many patients had not approached health services with their concerns due to “stay at home” guidance or a preference to avoid hospitals, which were seen as COVID-19 hot spots. This meant that health problems had likely worsened over time and would now require more attention:

But obviously there is a huge amount of people that just aren’t going to be seen that are just still waiting on a waiting list and our expectation is that you know there’s going to be new patients that are still gonna probably need to be referred so that when we are allowed to open up we are going to have a huge backlog to go through plus potentially more patients that are going to need to be seen afterwards and that is going to be a challenge within itself.

COV69, senior PT

These concerns and causes of distress were also present in the overall sentiment (Multimedia Appendix 4) of tweets shared about HCWs’ individual mental health. One of the key topics of concern was the exhaustion felt by both physicians and nurses, with some reporting feeling utterly shattered after several tough weeks working on the frontline looking after patients with COVID. Nurses shared reports of being signed off work with anxiety, whereas some physicians saw several family members admitted to COVID-19 wards and expressed anxiety at the young age of people being admitted. Other HCWs worried about exposure to COVID-19 during unpaid hospital secondments or placements and the risk of bringing COVID-19 home to their own families.

#### Organizational Support

HCWs acknowledged efforts to keep teams informed and promote good communication in the midst of things. However, particularly in the initial stages of the pandemic, guidelines were constantly changing, and staff received messages from multiple sources, which meant “things were blurry and confusing.” (COV97, consultant surgeon). Uncertainty resulted in anxiety and burnout from trying to keep up with new protocols:

It was really exhausting just trying to work out what we were doing with different things coming up in different places all the time. How we sort out our junior physicians, which junior physicians are staying, which are being redeployed? You know, and so on. Do we wear PPE? Do we not wear PPE?

COV45, consultant, gynecology

Student nurses, in particular, were concerned about the progress of their courses. Twitter was a platform for voicing angers and concerns:

QT@username: The silence from the NMC [Nursing and Midwifery Council] is deafening. We need clarity. Thousands of students across the country are full of anxiety waiting to hear what’s happening to them. We need guidance now@username; 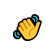 @username if we could have clarity for all #StudentNurses ASAP it would be great. HEI’s are taking different approaches Trusts are taking different approaches #PPEroulette We want to help, but we need your *clear* guidance. We need it now. #COVID19 #RCNStudents.

COVTweet835, student nurse

In some settings, work environments were more organized. HCWs suggested that enabling direct communication channels between hospitals helped to prepare for training and feel more confident, particularly communication with hospitals in countries that were ahead of the pandemic curve:

There was definitely a lot of planning which made everything feel very controlled. It made everything feel very calm. And it also made me feel, we felt like we knew what we were doing.

COV43, trainee general practitioner, accident and emergency services

Staff reported that strong leadership from management facilitated more compassion and awareness of mental health and well-being during the pandemic, which they would like to be continued in the future. Sentiment about the organizational support of HCW mental health was 23% negative, 70% positive, and 7% neutral. Some discussions focused on the problems that HCW staff (eg, locums and agency staff) were having access to accommodations close to hospitals they worked in, due to hotels and other accommodation closing because of lockdown restrictions and COVID-19 outbreaks. In addition, the NHS staff paid their respects to colleagues who had died of COVID-19. This led to discussions about poor staff access to PPE during the start of the pandemic, and the effects of this on staff mental health and morale. Between February and March 2021, some HCWs expressed relief in that more people were receiving their first dose of the COVID-19 vaccine, although they also warned that the United Kingdom government needed to take responsibility for failures earlier in the pandemic, as well as high levels of vaccination.

#### Social Network and Support

Participants highlighted family and friends as their main source of support and comfort during the peak of the pandemic, which gave them the strength to continue working in challenging conditions. The lack of social contact during the pandemic presented many challenges to some HCWs, who noticed a decline in their mental health, as many were unable to see their family and friends for a long period. However, this also encouraged participants to video call their family and friends for support to maintain their well-being.

Sentiment regarding support received from local social networks outside of work was 26% negative, 70% positive, and 4% neutral. Some HCWs mentioned having symptoms of post-COVID-19 condition (due to work-related infections) and being grateful for the support of colleagues and friends who helped them to pace clinical and nonclinical hours worked. Others mentioned the positive effect of working in COVID-19 vaccine clinics and interacting with supportive patients. Others shared positive tweets mentioning relief at being able to be vaccinated so that they could continue working safely:

I got the Oxford AZ. My husband, also a doctor, got the Pfizer. These were the ones available to us. We didn’t care about which one, rather “thank goodness we’re vaccinated!” The #vaccine 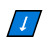 serious illness 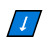 hospital admissions 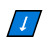 transmission All better than chancing COVID 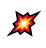
”

COVTwitter1277, NHS physician

Some HCWs mentioned worry about working antisocial hours and the prospect of burnout despite lower numbers of COVID-19 patients during the summer months. Relatives of HCWs also expressed concern for the health of those who could not work from home, citing examples of hospital pharmacists whose parents had died of hospital contracted COVID, and the stress felt by HCWs that they were putting their families at risk by working on the frontline.

#### Public and Government Support

Most participants expressed keen appreciation for the positive support conveyed by the public for their efforts during the pandemic. Many staff cited the generous donations of snacks, meals, toiletries, and other personal effects as genuinely helping sustain them during critical times. Food supplied to hospitals from restaurants and individual supporters was particularly welcomed, as it reduced the time needed for shopping or cooking and allowed more opportunities to rest after long and demanding shifts. Such outpourings of generosity were seen as buoying up the collective and individual morale, providing much-needed proof that the public appreciated their hard work:

The generosity of community at large outside of the hospital... the donations that we’ve had from food and all sorts of companies, to support from family and friends... and I think for me, like that’s what’s got me through this.

COV98, speech and language therapist

The “Clap for Carers” initiative was viewed as a more divisive public support response. Although many participants appreciated the gesture, some felt that it smacked of tokenism, particularly when it was juxtaposed against the flouting of social distancing rules and a seemingly blasé attitude toward the virus from some members of the public:

How can people go out, stand outside on balconies, clap for frontline people and then go out and break the rules?

COV86, speech and language therapist

Some participants also feared that public support would gradually wane once record-high waiting lists became a reality. Sentiment with regard to the perception of public support and government response was 46% negative, 48% positive, and 6% neutral. Sentiment toward the experience of access to PPE was 45% negative, 49% positive, and 6% neutral. In terms of negative perceptions of government support, some HCWs used Twitter to express their frustration at receiving just a 1% below-inflation pay rise despite the stress and trauma experienced while working on the frontline:

What a way to thank the people who’ve faced Covid straight in the eye & looked after the people suffering with it. Stayed strong to offer help to the people that are bereaved and scared. Whose job has changed so much, but we never complained. A 1% below inflation pay rise 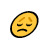.

COVTwitter1337, nurse practitioner

## Discussion

### Overview of Study

This study provided an overview of HCWs’ experiences of well-being during the first year of the COVID-19 pandemic. As found in the interviews and social media data, HCWs were willing and volunteered to work in unknown fields; however, redeployment generated anxiety mainly due to limited prior training and risk assessments and the barriers of adapting to a new working environment while wearing PPE. Well-being support varied across hospitals, and HCWs struggled to access it due to time constraints. In terms of mental health, mentions of feelings of trauma, PTSD, and anxiety were prominent. In addition to the challenges of redeployment, HCWs’ mental health was particularly affected by the copious amount of bad news on the media and at home and the fear of infecting their loved ones. The theme of organizational support exhibited dissonance in HCW experiences, with some praising strong leadership from management, whereas others explained how constantly changing guidelines caused additional anxiety.

### Redeployment

Similar to other studies in the United Kingdom, we found that redeployment generated anxiety for staff [4,36]. Our results did not capture the impact of redeployment on specific areas of the health care system, which is seemingly a major contributor to poor mental health in staff. However, other studies have consistently found that redeployment specifically to ICU units caused the most depressive symptoms and subsequently had the greatest impact on the mental health of HCWs in the United Kingdom [37,38]. This study showed evidence that during the COVID-19 pandemic, this was likely due to feeling ill equipped, receiving limited training, and having limited room for informal interactions within and outside clinical settings. Recent evidence supports this by indicating that the quantity and quality of training received before redeployment have a strong impact on HCW’s mental health [38].

### Preparedness and PPE Shortages

This study showed evidence that during the COVID-19 pandemic, this was likely due to feeling ill equipped, receiving limited training, and having limited room for informal interactions within and outside clinical settings. Recent evidence supports this by indicating that the quantity and quality of training received before redeployment have a strong impact on HCW’s mental health [38]. Other studies emphasize that feeling ill equipped is most strongly associated with shortages of PPE [39,40].

### Coping Strategies and Mental Health Support

As has been reported elsewhere, HCWs reported a wide range of mental health effects of delivering care during the pandemic [41-44]. These included PTSD, trauma, and exhaustion. It has been well documented that these feelings are more pronounced in ICU workers owing to high workloads, daily exposure to death, and irregular working hours [45]. Even in nonemergency situations, ICU workers experience the highest levels of anxiety compared with other units [46]. These results, together with those from our study, suggest that this line of research is relevant beyond the context of the pandemic, and there is sufficient information to move on to planning strategies to address this issue. These should include preventive mental health programs that have been proven to be successful and cost-effective that extend beyond the pandemic [47].

In terms of coping with these damaging mental health effects, HCWs’ perceptions of the support available to them were mixed. Similar to Schecter et al [48], some found sessions with clinical psychologists helpful, whereas others indicated that the well-being support offered at their hospitals did not align with their needs or work patterns. HCWs often appreciated and relied more on informal support from family friends and colleagues [25,49]. Muller et al [24] mirrored this idea by demonstrating that addressing organizational factors and support would improve mental health and well-being more than psychological help. In addition, some HCWs stated that they used their own methods to cope, such as mindfulness apps, with growing evidence that mindfulness-based apps improve well-being [50]. Meanwhile, in New York, physical activity appeared to be a popular coping mechanism [48]. Enhancing mental health and well-being training programs will equip HCWs with resilience-building tools, self-care, and coping strategies and empower them to seek support when needed.

### Organizational Support

A systematic review concluded that mental health problems in HCWs during COVID-19 correlated with organizational failures in the health care system [24]. Our results support this finding, as organizational support emerged as a main theme in discussions with HCWs. Mostly, HCWs deemed leadership from management as strong and that it facilitated compassion for their mental health and well-being. However, this was overshadowed by the constantly changing guidelines, which led to uncertainty. This has been confirmed by other studies [4], where uncertain and changing guidelines as well as the backlog of patient care caused superfluous cognitive burdens. A systematic review conducted by our team identified key principles for successful redeployment based on a compilation of strategies applied worldwide [51]. These included, for example, providing repeated shorter practical training sessions rather than receiving all information in 1 session, which is less flexible in adapting to emerging content and staff availability. In addition, some studies found that HCWs felt supported by hospital administration but not by their supervisors [36]. Our study provided insight into the excessive responsibilities of managers that prevented them from carrying out their roles as they would have liked, particularly in the case of nurses who were significantly understaffed. Future preparedness plans should include organizational policies that address psychological needs. Reviewing and improving the organization of care and a clearer sense of guidelines also reassured the staff.

### Social Network, Public, and Government Support

In terms of support from their social networks, the public, and the government, the HCWs were grateful and appreciative for their families and friends. The lack of socialization caused further damage to their well-being, as was seen in other populations during these times. It appears that social support is an integral element in maintaining HCWs’ mental health, as previous studies have found that a lack of social support affects sleep, anxiety, and stress [52]. In our previous related study [4], we also found that HCW placed significant emphasis on their responsibilities toward loved ones who consistently supported them and the invaluable support of the community. This was mirrored by Sun et al [53], who identified team support was a major protective factor against poor mental health during the pandemic. It is important to foster supportive networks in future pandemics by encouraging peer support and sharing experiences to mitigate the negative impact on mental health [54].

### Public Support

Most HCWs were appreciative of donations, meals prepared, and public support. Such outpourings of generosity were seen as buoying up collective and individual morale and positive feelings about the public appreciating their hard work. However, it was sometimes deemed tokenistic when HCWs compared their hardships with a large number of members of the public regularly breaking the COVID-19 guidelines. These mixed perceptions of public support were supported by sentiment analysis, which found an approximately equal number of positive and negative tweets on the topic. A topic that did not come up in this study was HCWs of Asian origin feeling discriminated against by the public [55]. This study perhaps did not pick up on this because of a lack of diversity within the sample, as most participants were of White British background.

### Deep-Rooted Concerns: Fears of Infecting Families

Qualitative research has shown that HCWs’ fear and anxiety of passing on COVID-19 to their families is a deep-rooted attitude [56], as it permeated all aspects of their descriptions of well-being. The damaging mental health effects that HCWs stated they felt were centered on the prospect of infecting others and being the cause of their distress. When discussing support from their social networks, this idea persisted, as participants mentioned that they could not fully reap the benefits of their loved ones’ support due to the anxiety of transmitting the virus. PPE shortages have generated concern in other studies because of the possibility of contracting COVID-19 and passing it on to family members [8,40]. This finding is not new, as it has been seen in past pandemics [57], emphasizing the need to include it as a priority in emergency response guidelines.

### Limitations

This study has several limitations. The HCWs included in the interview study worked in London, limiting the representation of experiences in other areas of the country and the generalizability of our findings. However, HCWs perceptions were captured through interviews and social media, which helped mitigate this limitation.

Although we collected data over 12 months, the last interviews were conducted in March 2021, limiting our exploration of experiences in the later stages of the pandemic. In this regard, 1 study concluded that anxiety levels in HCWs have increased since the pandemic was declared [58]. Although we included a broad sample of HCWs, future research is needed to conduct a more in-depth subgroup analysis to understand the specific experiences of each group.

Although purposive sampling was used to obtain a varied sample in terms of gender, role, and ethnicity, most interview participants were of White British backgrounds, and it is not possible to determine diversity for the social media sample. HCWs of different ethnic backgrounds are likely to have had different experiences and perceptions of sharing, and further research focusing specifically on their perspectives should be conducted. Although the influence of very active to passive Twitter users was not specifically controlled for, we did exclude retweets in our thematic analysis, where we instead focusing on the thematic content of the tweets than how many times they were retweeted or mentioned by other parties. However, the extent to which very “vocal” Twitter users, such as those with strong influence via the amount of their reach or followers was not controlled for in terms of influence on the overall data set, as suggested by Wojcik and Hughes [59]. Although the time of day at which the tweet is constructed can influence its sentiment—this was also not controlled for [60]—despite this limitation, it is also worth suggesting that any future studies that do control for more vocal actors—should additionally consider that the activities of HCWs in terms of hospital shift patterns that fit outside the “normal” work hours of the general population (either within the specific context of working on the frontline or generally)—may not always post in a similar spatiotemporal pattern or time of day as more regular users. Finally, in terms of sentiment analysis methods used in this paper—although the usage of more established sentiment analysis framework such as AFINN, BING, or NRC [61] is recognized as both relevant and useful—it was considered that these may not be as accurately applied to the rather unique specific public health emergency context of the mental health experiences HCWs working on the frontline during the COVID-19 pandemic. We do acknowledge that a comparative analysis of whether the use of these more established methods on a similar data set using the same search terms would be of great interest in future studies.

### Implications and Recommendations for Future Research

This study adds to the literature by addressing the knowledge gaps in the psychological reactions of the population during an infectious disease pandemic, which play a crucial role in determining not only the progression of the pandemic but also the well-being during and after peaks of infection. This is even more important to consider for HCWs, as they are at the forefront of the battle. The high prevalence of mental health problems and general burnout and exhaustion among HCWs has been repeatedly highlighted before and during the pandemic [62], with the World Health Organization officially acknowledging this risk to HCWs’ mental health, stating that additional long-term support must be provided to prevent symptoms of PTSD and depression [63]. Notwithstanding this, limited resources are allocated to HCWs’ broader well-being, as efforts focus mainly on mitigating potential clinical mental health problems.

The results of this study emphasize the need to consider HCWs’ personal perspectives to understand their context and barriers and facilitators to well-being, as well as to empower them to put into practice their own well-being strategies. Although previous studies have recommended tailored psychological interventions to manage symptoms and consolidate appropriate coping strategies, the results of this study imply that more systemic actions should be taken [20,64]. For instance, encouragement of HCW-led well-being initiatives, such as You ok and Doc? [65], Doctors Care [66], and EveryDoctor [67]. This could facilitate more open conversations between peers, sharing of experiences, and coping strategies outside of clinical settings, as well as empowering HCWs to inform the shape of health service provision. Another way to achieve this is by creating a more comfortable environment for HCWs and having adequate and appropriate equipment, most importantly, PPE; increased staff testing to ease the anxiety of transmitting the virus to family members could also address mental health and well-being concerns. As described earlier, these aspects are, for the most part, overlooked as necessary forms of staff well-being support that hospital administration, along with support from the government, should consider.

Future research should investigate personal factors that could potentially put some HCWs more at risk of damaging mental health and well-being effects. Yao et al [37] concluded that women, individuals who were married, and those who had engaged in <7 years of clinical work were more negatively affected by the COVID-19 pandemic. Wozniak et al [45] supported these conclusions but also demonstrated that assessments of symptoms of depression (9-item Patient Health Questionnaire and 7-item General Anxiety Disorder) and different types of anxiety were worse for those working in ICU versus those in non-ICU settings. In addition, although this study tried to include a broad sample of frontline HCWs, other hospital staff, such as professional services, security, and cleaning staff, who struggled with the immense workload and stress, were not possible to reach often because they are not direct NHS employees. Obtaining their perspectives is essential to fully understand the dynamics and perceptions of well-being in health care services.

### Conclusions

Our results provide a clear depiction of HCWs’ personal perspectives on their well-being needs, experiences, and strategies used to maintain well-being. Our findings highlight the need for a systemic approach broader than the current clinical focus on mental health problems. These findings should be used to inform current preventive strategies and future pandemic response guidelines and well-being funding allocation decisions. In addition, the innovative analysis method applied in this study yielded useful results for applied research in an efficient manner. This method is recommended for future emergency response research that considers big qualitative data in time-sensitive contexts [68].

## Appendix

Multimedia Appendix 1 Interview topic guide.

Multimedia Appendix 2 Boolean search terms.

Multimedia Appendix 3 Social media analysis coding framework.

Multimedia Appendix 4 Sentiment analysis.

Multimedia Appendix 5 Social media analysis: inclusion and exclusion criteria.

## Abbreviations

HCW: health care worker
ICU: intensive care unit
LISTEN: Collaborative and Digital Analysis of Big Qualitative Data in Time Sensitive Contexts
NHS: National Health Service
PPE: personal protective equipment
PTSD: posttraumatic stress disorder
RREAL: Rapid Research, Evaluation and Appraisal Lab
