# Supplementary material for: Frontline Health Care Workers’ Mental Health and Well-Being During the First Year of the COVID-19 Pandemic: Analysis of Interviews and Social Media Data
Source: J Med Internet Res. 2023 Aug 14;25:e43000. doi: 10.2196/43000 (PMC10426381; doi:10.2196/43000)
Supplement: Multimedia Appendix 1 [file jmir_v25i1e43000_app1.docx]

**Appendix 1: INTERVIEW TOPIC GUIDE: HEALTHCARE WORKERS (HCWs)**

Date:

Interviewee ID code:

Time interview started:

Time interview stopped:

Name of interviewer:

Digital recording code:

Venue (eg. telephone call, Skype):

General comments and observations:

**Respondent** Information

| Gender | Age | Time in service (mm/yy) | Education level | Role/position | Sector and type of facility | Location of facility |
| --- | --- | --- | --- | --- | --- | --- |
|  |  |  |  |  |  |  |

**First, I want to ask you about your work and the services you provide. (This section not COVID-19 specific).**

1. **Background**

- *Can you tell me about your role?*
- *Please briefly describe your normal daily tasks/responsibilities.*
- *What do you enjoy about your work? (this question is to ensure starting on the upbeat)*
- *What are the main of the challenges you face in your work? (probes: policy, service delivery, etc.)*
- *What health services can patients receive from you?*

1. **Can you tell me about how your work is coordinated?**

- *What connections and coordination to you have with health managers and other healthcare providers?*
- *How is your work supervised? How often? By whom? What responsibilities do you have to supervisor, and does supervisor have to you? Is the supervision supportive? (here need to check if they are actually supervised supportively or just meet a supervisor to hand in data, M&E).*

**Now I want to ask you about health services during COVID-19 outbreak.**

1. **Have you been in contact with COVID-19+ patients?**

*Probes:*

- - *In what capacity?*
  - *What areas of the hospital?*
  - *How have you responded to these patients?*
  - *What impact did this have on your daily work?*
  - *What psychological/emotional impact did this have on you?*

1. **Has the COVID-19 outbreak affected health services at your health facility?**

*Probes:*

- *Managing case isolation and non-COVID patients in an appropriate way.*
- *Capacity of staff to make correct diagnoses and act on them.*
- *Ability to juggle COVID management tasks with regular tasks.*
- *Impact of COVID-19+ care delivery on the delivery of services to non-COVID-19+ patients (i.e. cancellation of elective surgeries)*
- *Supply of drugs, supplies, equipment and PPE?*
- *Redeployment of staff*
- *Supervision?*
- *Completing and sending reports and/or lab samples/results?*
- *Care seeking by the population? E.g. The ability or willingness of patients to comply with referrals?*
- *Motivation and capacity of staff to work? (probe: exhaustion, fear, etc.)*
- *Collecting data?*
- *Psychosocial and moral support – did they receive any? Enough?*
- *Others?*

**5. What were the preparedness strategies implemented at your local Trust?**

*- Were you involved in decision-making*

*- Did you feel these strategies were enough?*

*- Should the Trust have prepared differently?*

*-Did you receive any training? (including but not limited to PPE training?)*

**6. Do you currently have any concerns or fears in relation to current response efforts?**

**7. Which activities are HCWs able to do effectively and which are less effective? Why?**

- *What do you think about the role of HCWs during the outbreak? What influenced their performance? (probes: training, experience, status in community).*
- *Who coordinated their activities? How? What can we learn from these? How could it be improved?*
- *What are the challenges in doing the activities as requested? What?*
- *How do you feel about HCWs ability to respond to the needs of the community? (COVID-19 and other routine services)*
- *How did local communities respond to HCWs during the outbreak? Why do you think this was?*

**8. Are you using local data to inform response efforts?**

- What type of data?

- Who normally collects the data?

-How are data shared?

-How are data used to make changes in practice?

**9. How have health services been strengthened/ can be strengthened during the outbreak?**

*Probes:*

- *Policies? e.g. Guidance and emergency protocols?*
- *Guidance on how to maintain essential services during an outbreak. What would help HCWs to maintain normal services as well as COVID related services?*
- *Health promotion and community engagement. Probe. How?*
- *Linkage to other support organisations, e.g. Care/referral for children?*
- *Support to HCWs from the health system and partners?*
- *In future outbreaks how can HCWs contribute to enhancing trust in the health system?*
- *Supporting, advising communities. How? Who else can collaborate on that? Who do communities trust?*
- *Capacity for rapid response*
- *Support to HCWs from the health system and partners?*

**10. What do you think should be changed to make health services more effective in future emergencies?**

*Probes:*

- *Coordination of COVID-19 response.*
- *Early detection and reporting.*
- *On-going health promotion. E.g. potential sources of infection, safe practice?*
- *Mobilisation? E.g. identifying and coordinating trusted community volunteers and support?*
- *Policies?*
- *Action plans and protocols.*
- *Support to HCWs? From whom? How?*
- *Community education and mobilization strategies?*
- *Disease outbreak control activities?*

**Thank you for your time and for sharing your opinions and experiences with us.**
