# Supplementary material for: Frontline Health Care Workers’ Mental Health and Well-Being During the First Year of the COVID-19 Pandemic: Analysis of Interviews and Social Media Data
Source: J Med Internet Res. 2023 Aug 14;25:e43000. doi: 10.2196/43000 (PMC10426381; doi:10.2196/43000)
Supplement: Multimedia Appendix 2 [file jmir_v25i1e43000_app2.docx]

### Appendix 2: Boolean search terms

#### Mental health effects

((bio:"healthcare professional" **OR** bio:"healthcare worker" **OR** bio:"doctor" **OR** bio:"GP" **OR** bio:"Pharmaci*" **OR** bio:"Radiograph*" **OR** bio:"Therap*" **OR** bio:"Neurolo*" **OR** bio:"Pyschol*" **OR** bio:"clinic*" **OR** bio:"nurse" **OR** bio:"physio*" **OR** bio:"midwi*" **OR** bio:"obstet*" **OR** bio:"geriatr*") **AND** ("coronavirus" **OR** "#coronavirus" **OR** “corona” **OR** "COVID-19" **OR** "COVID 19" **OR** "COVID19" **OR** "#COVID19" **OR** "COVID_19" **OR** "COVID" ) **AND** ("redeploy*" **OR** "stress*" **OR** "overwork*" **OR** "face-to-face" **OR** "face to face" **OR** "anxiet*" **OR** "anxi*" **OR** "scared" **OR** "afraid" **OR** "tired" **OR** "trauma*" **OR** "burn* out" **OR** "burnout" **OR** "not able" **OR** "insomni*" **OR** "sad*" **OR** "bad news" **OR** "mental health" **OR** "fright*" **OR** "worr*" **OR** "scared" **OR** "afraid" **OR** "tired" **OR** "alcho*" **OR** "sleep*" **OR** "rest*" **OR** "drink*" **OR** "alcohol*" **OR** "drug*" **OR** "smok*" **OR** "dying*" **OR** "death*" **OR** "personal" **OR** "pending" **OR** "expect*" **OR** "detach*" **OR** "autopilot" **OR** "auto pilot" **OR** "cope*" **OR** "coping" **OR** "responsi*" **OR** "access*" **OR** "support*" **OR** "friend*" **OR** "adequa*" **OR** "vacci*" **OR** "lockdo*" **OR** "first wave*" **OR** "risk*" **OR** "unfamilia*" **OR** "second wave*" **OR** "firstwave*" **OR** "secondwave*") )

#### Organisational

((bio:"healthcare professional" **OR** bio:"healthcare worker" **OR** bio:"doctor" **OR** bio:"GP" **OR** bio:"Pharmaci*" **OR** bio:"Radiograph*" **OR** bio:"Therap*" **OR** bio:"Neurolo*" **OR** bio:"Pyschol*" **OR** bio:"clinic*" **OR** bio:"nurse" **OR** bio:"physio*" **OR** bio:"midwi*" **OR** bio:"obstet*" **OR** bio:"geriatr*") **AND** ("coronavirus" **OR** "#coronavirus" **OR** “corona” **OR** "COVID-19" **OR** "COVID 19" **OR** "COVID19" **OR** "#COVID19" **OR** "COVID_19" **OR** "COVID" ) **AND** ("at work" **OR** "hospital" **OR** "clinic*" **OR** "ward*" **OR** "ICU*" **OR** "unit*" **OR** “NHS trust*”) **AND** ("co-worker*" **OR** "co worker*" **OR** "communi*" **OR** "comms" **OR** "colleague*" **OR** "team*" **OR** "group*" **OR** "wellbeing" **OR** "leader*" **OR** "staff*" **OR** "dynam*" **OR** "manag*" **OR** "redploy*" **OR** "deploy*" **OR** "access*" **OR** "unfair*" **OR** "mental health*" **OR** "mentalhealth*" **OR** "ppe*" **OR** "clear*" **OR** "clarity" **OR** "anxi*" **OR** "worr*" **OR** "guide*" **OR** "polici*" **OR** "policy*" **OR** "compulso*" **OR** "voluntar*" **OR** "duties*" **OR** "duty" **OR** "profression*" **OR** "organisat*" **OR** "manag*" **OR** "upskill*" **OR** "skill*" **OR** "network*" **OR** "abilit*" **OR** "develop*" **OR** "remote*" **OR** "isolat*"))

#### Social network/ support network

((bio:"healthcare professional" **OR** bio:"healthcare worker" **OR** bio:"doctor" **OR** bio:"GP" **OR** bio:"Pharmaci*" **OR** bio:"Radiograph*" **OR** bio:"Therap*" **OR** bio:"Neurolo*" **OR** bio:"Pyschol*" **OR** bio:"clinic*" **OR** bio:"nurse" **OR** bio:"physio*" **OR** bio:"midwi*" **OR** bio:"obstet*" **OR** bio:"geriatr*") **AND** ("coronavirus" **OR** "#coronavirus" **OR** “corona” **OR** "COVID-19" **OR** "COVID 19" **OR** "COVID19" **OR** "#COVID19" **OR** "COVID_19" **OR** "COVID" ) **AND** ("family*" **OR** "friend*" **OR** "support*" **OR** "relationsh*" **OR** "interact*" **OR** "help*" **OR** "group*" **OR** "partner*" **OR** "daughter*" **OR** "wife*" **OR** "husband*" **OR** "boyfriend*" **OR** "bff" **OR** "girlfriend*" **OR** "son*" **OR** "mum*" **OR** "mother*" **OR** "father*" **OR** "mate*" **OR** "dad*" ))

#### Wider context

((bio:"healthcare professional" **OR** bio:"healthcare worker" **OR** bio:"doctor" **OR** bio:"GP" **OR** bio:"Pharmaci*" **OR** bio:"Radiograph*" **OR** bio:"Therap*" **OR** bio:"Neurolo*" **OR** bio:"Pyschol*" **OR** bio:"clinic*" **OR** bio:"nurse" **OR** bio:"physio*" **OR** bio:"midwi*" **OR** bio:"obstet*" **OR** bio:"geriatr*") **AND** ("coronavirus" **OR** "#coronavirus" **OR** “corona” **OR** "COVID-19" **OR** "COVID 19" **OR** "COVID19" **OR** "#COVID19" **OR** "COVID_19" **OR** "COVID" ) **AND** ("public*" **OR** "support*" **OR** "response*" **OR** "government*" **OR** "measures" **OR** "disillus*" **OR** "accommodat*" **OR** "living arrangement*" **OR** "government respons*" **OR** "government measure*" **OR** "impact*" **OR** "public support*" **OR** "clapforcarers*" **OR** "clap for carers*" **OR** "clap-for-carer*" **OR** "rainbow*" **OR** "nhs rainbow*" **OR** "childcarei*"))

#### PPE

((bio:"healthcare professional" **OR** bio:"healthcare worker" **OR** bio:"doctor" **OR** bio:"GP" **OR** bio:"Pharmaci*" **OR** bio:"Radiograph*" **OR** bio:"Therap*" **OR** bio:"Neurolo*" **OR** bio:"Pyschol*" **OR** bio:"clinic*" **OR** bio:"nurse" **OR** bio:"physio*" **OR** bio:"midwi*" **OR** bio:"obstet*" **OR** bio:"geriatr*") **AND** ("coronavirus" **OR** "#coronavirus" **OR** “corona” **OR** "COVID-19" **OR** "COVID 19" **OR** "COVID19" **OR** "#COVID19" **OR** "COVID_19" **OR** "COVID" ) **AND** ("PPE*" **OR** "ppe*") **AND** ("physical" **OR** "breath*" **OR** "hot*" **OR** "anxiet*" **OR** "scared" **OR** "afraid" **OR** "tired" **OR** "abilit" **OR** "access*" **OR** "ramadan" **OR** "eid" **OR** "eid al-adha" **OR** "dhydrat*" **OR** "hydrat*" **OR** "slow*" **OR** "headach*" **OR** "mood*" **OR** "emotion*" **OR** "worr*" **OR** "perform*" **OR** "usual*" **OR** "tired" **OR** "wellbeing" **OR** "worr*" **OR** "sweat*" **OR** "group*" **OR** "professi*" **OR** "comms*" **OR** "communic*" **OR** "speak*" **OR** "hear*" **OR** "clear*" **OR** "clinic*" ))
