# Supplementary material for: Frontline Health Care Workers’ Mental Health and Well-Being During the First Year of the COVID-19 Pandemic: Analysis of Interviews and Social Media Data
Source: J Med Internet Res. 2023 Aug 14;25:e43000. doi: 10.2196/43000 (PMC10426381; doi:10.2196/43000)
Supplement: Multimedia Appendix 3 [file jmir_v25i1e43000_app3.docx]

## Appendix 3: Social media analysis coding framework

| **1) Individual mental health** | **2) Organisational** | **3) Social network/ support network** |
| --- | --- | --- |
| - Mental health effects (Trauma from striking experiences; Loss of sleep; Anxiety; Drinking [61]; smoking; drug use; Sadness of number of deaths; Impact [35] of patient demographics).  - Worries about what’s coming (Backlog of non-emergency procedures; Increased worry after 1st wave; Loss of hope).  - Detachment (working on automatic pilot)  - Coping strategies - Mental health support use preferences  - Clinical work/sense of duty (Sense of duty vs. Risk and capacity; Overwork or lack thereof; Sadness; Professional identity).  - Redeployment experiences (New settings; new work; Returning to previous role; Differences between professions) | - Group dynamics (Positives; Leadership; Communication; Differential treatment)  - Management and new ways of working (Anxiety over clarity; Differences between professionals)  - Positives (Upskilling; Developing new networks; Remote working) | - Relationships outside work  - Family and close friends |
| **4) Wider context (*potential ambiguity)** | **5) PPE** |  |
| - Public support  - Government response  - Living arrangements | - Individual (Physical impact / impact on morale; Clinical work)  - Organisational (Availability and accessibility; Group dynamics- Communication with PPE) |  |
