# Supplementary material for: Frontline Health Care Workers’ Mental Health and Well-Being During the First Year of the COVID-19 Pandemic: Analysis of Interviews and Social Media Data
Source: J Med Internet Res. 2023 Aug 14;25:e43000. doi: 10.2196/43000 (PMC10426381; doi:10.2196/43000)
Supplement: Multimedia Appendix 4 [file jmir_v25i1e43000_app4.docx]

### Appendix 4: Sentiment analysis

**Sentiment analysis criteria for mental health and wellbeing of HCWs**

HCW experience of mental health and wellbeing during Covid-19 pandemic

**Definition & Context:**
We aim to gather accounts of the experiences of healthcare workers (HCWs) in the challenges and constraints they might have to their mental health and wellbeing during the COVID-19 pandemic.

**Sentiment analysis of mental health and wellbeing**

**Positive (P)**

- Post communicating overall trust and satisfaction with experience of or guidelines and support for mental health and wellbeing in the context of the COVID-19 pandemic
- Posts are affirming of mental health and wellbeing delivery and experiences of staff

**Negative (N)**

- Post contains negative attitude/arguments against Covid-19 treatment / guidelines / support / of HCW mental health and wellbeing
- Post discourages the following of recommended treatment / guidelines / support related to mental health and wellbeing
- Post shares bad HCW experiences of working on Covid frontline and the effect of this on mental health and wellbeing.

**Neutral (NT)**

- Post contains no elements of uncertainty, positive or negative content.
- Post contains general statement(s) or link(s) to item(s) (e.g., news articles/papers) with no expression of sentiment.
- Post includes factual statements/recommendations about COVID-19 and mental health and wellbeing, but no other sentiment.
