# Supplementary material for: Frontline Health Care Workers’ Mental Health and Well-Being During the First Year of the COVID-19 Pandemic: Analysis of Interviews and Social Media Data
Source: J Med Internet Res. 2023 Aug 14;25:e43000. doi: 10.2196/43000 (PMC10426381; doi:10.2196/43000)
Supplement: Multimedia Appendix 5 [file jmir_v25i1e43000_app5.docx]

## Appendix 5: Social media analysis: Inclusion and exclusion criteria

| **Inclusion Criteria Definition** |
| --- |
| Content refers specifically to the experience of HCWs within the overall topic of mental health and wellbeing as covered in the interview analysis. I.e., People actually talking about wellbeing topics, rather than things we infer might affect wellbeing (without them mentioning it). |
| Example of tweet to include: "Government is not supporting healthcare workers." We would include this because it mentions explicitly about HCWs, something that is affecting HCWs (and therefore their wellbeing) directly. |
| **Exclusion Criteria Definition** |
| Content does NOT refer specifically to the experience of HCWs within the overall topic of mental health and wellbeing. |
| Example to exclude: "I am against government budget cuts". This is something that ultimately probably affects healthcare workers, but there's no explicit mention to them or their wellbeing. Even if it's a HCW writing it, we can't know if they're against government cuts because of something work/wellbeing-related |
